# Maternal perception of fetal movements: Views, knowledge and practices of women and health providers in a low-resource setting

**DOI:** 10.1371/journal.pgph.0000887

**Published:** 2023-03-29

**Authors:** Katinka Weller, Natasha Housseine, Rashid S. Khamis, Tarek Meguid, G. Justus Hofmeyr, Joyce L. Browne, Marcus J. Rijken

**Affiliations:** 1 Department of Obstetrics and Gynecology, Division of Obstetrics and Fetal Medicine, Erasmus MC University Medical Centre Rotterdam, Rotterdam, The Netherlands; 2 Julius Global Health, Julius Centre for Health Sciences and Primary Care, University Medical Centre Utrecht, Utrecht, The Netherlands; 3 Division of Woman and Baby, University Medical Centre Utrecht, Utrecht, The Netherlands; 4 Department of Obstetrics and Gynaecology, Mnazi Mmoja Hospital, Zanzibar, Tanzania; 5 School of Health and Medical Sciences, The State University of Zanzibar, Zanzibar, Tanzania; 6 Global Health Section, Department of Public Health, University of Copenhagen, Copenhagen, Denmark; 7 Child Health Unit, Department of Paediatrics and Child Health, University of Cape Town, Cape Town, South Africa; 8 Department of Maternal & Child Health, Medical School, University of Namibia, Windhoek, Namibia; 9 Effective Care Research Unit, Eastern Cape Department of Health, University of the Witwatersrand/Walter Sisulu University, East London, South Africa; 10 Obstetrics and Gynecology Department, University of Botswana, Gaborone, Botswana; UP Manila: University of the Philippines Manila, PHILIPPINES

## Abstract

The study assessed perception, knowledge, and practices regarding maternal perception of fetal movements (FMs) among women and their healthcare providers in a low-resource setting. Semi-structured interviews, questionnaires and focus group discussions were conducted with 45 Zanzibar women (18 antenatal, 28 postpartum) and 28 health providers at the maternity unit of Mnazi Mmoja Hospital, Zanzibar, Tanzania. Descriptive and thematic analyses were conducted to systematically extract subthemes within four main themes 1) knowledge/awareness, 2) behavior/practice, 3) barriers, and 4) ways to improve practice. Within the main themes it was found that 1) Women were instinctively aware of (ab)normal FM-patterns and healthcare providers had adequate knowledge about FMs. 2) Women often did not know how to monitor FMs or when to report concerns. There was inadequate assessment and management of (ab)normal FMs. 3) Barriers included the fact that women did not feel free to express concerns. Healthcare providers considered FM-awareness among women as low and unreliable. There was lack of staff, time and space for FM-education, and no protocol for FM-management. 4) Women and health providers recognised the need for education on assessment and management of (ab)normal FMs. In conclusion, women demonstrated adequate understanding of FMs and perceived abnormalities of these movements better than assumed by health providers. There is a need for more evidence on the effect of improving knowledge and awareness of FMs to construct evidence-based guidelines for low resource settings.

## Introduction

Effective interventions are needed to meet the Sustainable Development Goals and The Every Newborn Action Plan for newborn mortality and stillbirth reduction (≤12 per 1000 births by 2030), particularly in middle- and low-income countries where the burden is greatest [1]. A range of simple to sophisticated methods to detect problems during pregnancy and birth have been developed [2]. Fetal movements (FMs) are the oldest and most basic form of fetal monitoring and can be done by the mother herself without any resources. The complexity of movements requires fetal neuromuscular development and a normal metabolic state of the central nervous system [3]. Women, on average, start to feel movements between 18–20 weeks of gestation and will quickly be aware of a regular, personal pattern of movements of their child [4, 5]. The amount of daily movements increase during the third trimester and reach a plateau in the last six weeks of pregnancy. Observational evidence indicates that reduced body and breathing movements are appropriate physiological responses to conserve energy during hypoxia or acidemia [6]. Reduced movements could precede stillbirth before any other recordable fetal changes, and are registered from as early as two weeks up to 48 hours before the confirmation of fetal death [7–11]. This suggests that early detection and management of reduced FMs may prevent adverse perinatal outcomes [7–10]. However there is lack of evidence for interventions that improve FM awareness and management of reduced FMs from randomised trials to guide clinical practice [12–14]. Limitation of these randomised trials is that reporting decreased fetal movements is an intuitive action which cannot be eliminated from the control group and the lack of generalizability of the trials, which have all been conducted in high-income countries, to resource-constrained settings with high burden of stillbirths and different contextual realities [15]. There is a dearth of evidence on women’s and healthcare provider’s awareness, knowledge, subsequent practices and use of FMs as a strategy to prevent stillbirths in low-resource settings, hence it is difficult to develop appropriate contextualised interventions for these settings. Notably, there is lack of evidence on how women in low-resource settings use this vital sign to monitor the wellbeing of their baby and how they report reduced FMs to healthcare providers. Likewise, it is also unclear how healthcare providers educate women about FM monitoring and the health system response to women who report reduced FMs. This could be a missed opportunity, especially in these settings since monitoring FMs does not require any resources. Therefore, this study aimed to explore knowledge and perspectives on maternal perception of FMs and practices on perceived abnormal fetal movements among mothers and healthcare providers in a busy maternity unit in a low-income country.

## Methods

### Ethics statement

The study was approved by the Zanzibar Medical Research and Ethics Committee in August 2017 (Protocol no: ZAMREC/0004/AGUST/17). Written informed consent was obtained from all participants, interviews were voluntary and participants were able to decline participation at any time. All participants agreed for their quotes to be published anonymously.

### Study design

This study was embedded within a large prospective study that aimed to develop a screening tool to identify labouring women at risk of perinatal death as they were being admitted to the labour ward [16]. Assessment of maternal perception of fetal movement was an important component of this tool in which research assistants used a newly-developed questionnaire to ask women about their baby’s movement. The larger study, however, was an observational study and so no FM education or other intervention was given to participants [16]. This part of the study used a mixed-method approach to provide a comprehensive and in-depth understanding of the topic within the contextual limitations. Semi-structured interviews were conducted with women and antenatal care (ANC) nurses; focus group discussions (FGDs) supplemented with questionnaires were used among skilled birth attendants. This study was reported according to the COREQ checklist (S1 Text) [17].

### Setting

This study was conducted in the maternity unit of Mnazi Mmoja Hospital (MMH), the government-run referral hospital on the Zanzibar archipelago that caters for 11.000–13,000 births annually [18]. It was government policy that maternal healthcare services throughout pregnancy, childbirth and puerperium are provided free of charge, however, women spent out of pocket for laboratory investigations and to buy medicine and supplies that were not available in the hospital. The facility-based stillbirth rate at MMH was 39 per 1000 total births, around half of which occurred intrapartum [19, 20]. The maternity unit consisted of a maternity ward with one admission room, three labour room with about eight beds each, three individual delivery rooms, and two ANC clinics. The regular ANC was run by nurses. The obstetric ANC functioned as referral clinic for women with complications during pregnancy and was operated by registrar doctors.

### Participant selection

Participants in both the antenatal and postpartum period were interviewed to elicit women and healthcare providers’ experiences and practices with FMs throughout pregnancy and labour care (including the time of admission). Between October 2017 and February 2018, all nurse-midwives and doctors (registrars and intern doctors) of the department were invited to participate in a questionnaire and FGD which took place outside working hours. Separate FGDs were organised for nurse-midwives and doctors of equal seniority to allow freedom of expression within their cadre and divergent of views. In addition, purposive sampling was used to select pregnant women who were ≥18 years and ≥18 weeks gestational age for one-time semi-structured interviews. Women were approached and interviewed at the ANC clinic (either routine or obstetric clinic) before or after their consultation with the healthcare provider. Also, postpartum women were recruited before hospital discharge using purposeful sampling to include a relatively equal number of women with and without adverse perinatal outcomes (stillbirth, neonatal death and/or Apgar score <7 at 5 minutes). Pregnant and postpartum women were approached and asked to participate in an interview by a Kiswahili speaker, either a female intern doctor or a female researcher with a diploma in psychology and KW. Women were greeted and given information about the study including the purpose of the study, the process of interview, the voluntary nature of their participation and confidentiality of the data collected. Privacy was ensured by interviewing women in private rooms or spaces.

### Data collection

The researchers developed a questionnaire and interview- and FGD-guidelines to explore the main themes (S2 Text): awareness and knowledge, behavior and practices, barriers and opportunities for improvements in the usage of maternal perception of FMs for fetal surveillance. These themes were selected in advance by the authors after extensive consideration, reflection, and discussion in order to fulfill the aim of this study. The questionnaire was completed by all healthcare providers prior to the start of the FGDs with the aim of having objective answers before starting the FGDs. Answers given in the questionnaires were anonymously used to guide the discussions. Behavior and practices around FMs were assessed at three distinct time points: during antenatal care, on admission to the labour ward and intrapartum. Data collection tools were translated to Kiswahili and pilot tested. Trained junior researchers outside the department of obstetrics conducted the interviews and FDGs to allow participants to freely share their knowledge and opinions. A Kiswahili native speaker, either a female intern doctor or a female researcher with a diploma in psychology, conducted the antepartum and postpartum interviews. The interviewers and participant did not know each other prior to the interviews. A male intern doctor and native speaker (RSK) with prior experience in the maternity unit and in moderating FGDs mediated the FGDs. The role of the moderator during the FGDs was to guide the course of the discussion and encourage participation by all, for example by creating a relaxed and friendly environment for participants to give their honest opinions and give everybody the opportunity, one-by-one to speak and ask questions. The local interviewers/moderator worked closely with KW. KW was a Dutch medical student who spent a total of six months in the study setting during which she assisted with research activities, attended clinical meetings but had no clinical involvement at the maternity unit. KW was present during all interviews and FGDs and assisted the interviewers/moderator to adequately explore topics and emerging findings during data collection. NH was a PhD student and supervised the study. She had been working as a doctor at the maternity unit of MMH at the time of the study for about three years. Antepartum and postpartum interviews lasted 15–30 minutes and 5–20 minutes respectively, while the FDGs with staff members lasted 40–90 minutes. Recruitment of participants continued until saturation of information was reached. Interviews were translated immediately to English and detailed field notes were written down during interviews, both in Kiswahili and English. FGDs were audio recorded with the permission of participants and afterwards transcribed and translated by RSK. Transcripts were not returned to participants. Multiple-choice questionnaires were anonymously self-administered and completed by the health providers prior to the FGDs (S3 Text).

Sociodemographic characteristics (age, marital status, education, occupation and obstetric history), perinatal outcomes and cross-validation of answers given in the interviews of participating women were collected from participants, ANC cards, hospital files and, if necessary, from data of the main study.

### Data analysis

Qualitative data was analyzed using thematic analysis with the major themes of interest: knowledge/awareness, behavior/practice, barriers, and improvements. Data of the FGDs were analyzed using whole groups (i.e., nurse-midwives and doctors). After familiarization with FGDs and interviews, open coding was conducted by KW using the software MAXQDA version 12. Codes were analyzed for patterns and systematically combined into subthemes. KW conducted the main analysis and discussed with NH and RSK for their local perspective on the interpretation of participants views and behavior. In addition, quotes are presented anonymously as either from antepartum women, postpartum women or healthcare providers to illustrate common views and support our interpretation. Questionnaires were entered into a KoBoToolbox electronic database. Descriptive analysis was performed on quantitative data using Microsoft Office Excel 365 ProPlus with the aim of substantiating the qualitative data.

## Results

### Baseline characteristics

Eighteen women in the ANC clinics, 28 post-delivery women and three ANC nurses were interviewed. There were no women that declined to participate in this study. In addition, 25 skilled birth attendants (six registrar doctors and 19 nurse-midwives) participated in four FGDs; one group with six registrars and three groups with nurse-midwives (consisting of five, eight and six participants). Hence, most skilled birth attendants in the maternity unity participated in the study. The health providers also completed the questionnaire (n = 28). Characteristics of women and health providers are summarised in Table 1. The median age for ANC and postpartum women was 28.3 [Interquartile range (IRQ): 19–41] and 26.2 [IQR: 19–37] years respectively. The majority of participating women at least finished primary education and were unemployed (housewives). Gravidity and parity were equal in both groups of women. Due to purposeful sampling, half of the postpartum women experienced adverse perinatal outcomes. The majority of staff members had one to five years of experience in labour care (Table 1). As expected, the nurse-midwives performed admission assessment of women in labour.

**Fig 1:**
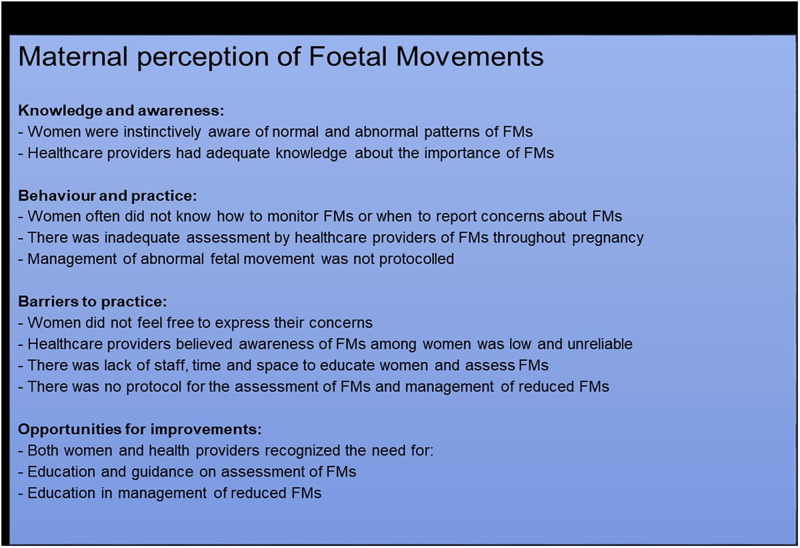
Themes for maternal perception fetal movement.

An overview of themes and results is presented in Fig 1.

**Table 1:** Background characteristics women and staff participants in this study.

	**ANC Women (n = 18)**	**Postpartum women (n = 28)**
Age (years)	28.3 [19–41]	26.2 [19–37]
Gravidity	3 [1–5]	3 [1–7]
Parity	2 [0–4]	2 [0–6]
Living children	1 [0–4]	2 [0–5]
Abortions/miscarriages	0 [0–3]	0 [0–2]
Stillbirths	1 [0–1]	0 [0–1]
Gestational age at interview (ANC) (months)	7 [6–10]	-
Gestational age at delivery (weeks)	-	35 [28^+0^–42^+2^]
Outcome of pregnancy		
Healthy baby	-	14 (50.0)
Adverse perinatal outcome	-	14 (50.0)
Stillbirth Fresh	-	4 (28.6)
Stillbirth Macerated	-	3 (21.4)
Low Apgar (<7 at 5 min)	-	4 (28.6)
Neonatal death		3 (21.4)
Current marital status		
Unmarried	1 (5.6)	1 (3.7)
Married	17 (94.4)	26 (96.3)
Highest level of education		
No education	1 (5.6)	1 (3.7)
Primary school	3 (16.7)	5 (18.5)
Secondary school	13 (72.2)	16 (59.3)
Tertiary school/ University	1 (5.6)	5 (18.5)
Occupational status		
Unemployed(housewife)	10 (55.6)	19 (70.4)
Employed	2 (11.1)	3 (11.1)
Self-employed	6 (33.3)	5 (18.5)
Characteristics of healthcare providers		
	**Registrars (n = 6)**	**Nurse-midwives (n = 22)**
Years of experience in labour care		
< 1	1 (16.7)	9 (41.0)
1–5	4 (66.7)	10 (45.5)
5–10	1 (16.7)	0 (0.0)
> 10	0 (0.0)	3 (13.6)
No of days a week working at the admission room or in the ANC clinic		
Never	4 (66.7)	0 (0.0)
< 1	2 (33.3)	5 (22.7)
1–3	0 (0.0)	6 (27.3)
> 4	0 (0.0)	11 (50.0)

Values are presented as number (%) or median [range].

### Knowledge and awareness

#### Variety in describing perceived FMs

Women used a variety of Kiswahili vocabulary to describe movements they experienced: ‘*anacheza’* (it plays), *‘anapiga’* (it kicks), ‘*anazunguka*’ (it rotates), ‘*anatembea’* (it walks) and ‘*anaogelea’* (it swims), ‘*anasukuma’* (it pushes) and *‘anatwita’* (it beats/flicks). Healthcare providers considered fetal movements as kicks, rolling, hiccups or playing of the fetus (Table 2).

**Table 2:** Healthcare providers responses to questionnaire on fetal movement awareness, knowledge and practices (n = 28).

Questions (multiple answers possible)		Responses n (%)
***What are fetal movements?***	Kicks of the fetus	14 (50.0)
	Rolls of the fetus	10 (35.7)
	Kick, rolls and hiccups of the fetus	5 (17.8)
	Other: *‘Playing of the child’*	2 (7.1)
	Hiccups of the fetus	0
***How often do you ask women about fetal movements in the ANC*, *on admission or in the labour ward?***	Almost always	20 (71.4)
	Often	8 (28.6)
	Sometimes	0
	Seldom	0
	Never	0
***How do you assess fetal movement?***	Asking the woman	22 (78.6)
	Palpation of the abdomen	11 (39.3)
	Ultrasound scan	11 (39.3)
	Check ANC card	2 (7.1)
	I never assess fetal movement	0
***What*, *in your opinion*, *do fetal movements tell us?***	Health of the fetus	22 (78.6)
	Whether or not the mother pays attention to the child	6 (21.4)
	None of the above	3 (10.7)
	Other: *‘The life of the baby’*	1 (3.6)
	Whether or not the fetus is asleep	0
	I don’t know	0
***What is change in fetal movement?***	Change in frequency of movements	18 (64.3)
	Change in strength of movements	13 (46.4)
	I don’t know	11 (39.3)
	Other	0
***Which of the following changes in fetal movements do you think need extra attention?***	Decreased fetal movements	23 (82.1)
	Absent fetal movements	15 (53.6)
	Increased fetal movements	13 (46.4)
	Sudden vigorous fetal movements	11 (39.3)
	I don’t know	0
***How do you manage a woman who presents with changes in fetal movements***	Clarify when she last felt the baby move, check FHR and do an ultrasound	16 (57.1)
	Routine examination on admission	15 (53.6)
	Clarify when she last felt the baby move and check FHR	11 (39.3)
	I don’t know	1 (3.6)
***Around what gestational age do you start asking women about fetal movements?***	Around 18–20 weeks	27 (96.4)
	Around 28–30 weeks	6 (21.4)
	Around 10–12 weeks	0
	I don’t know	0
***What do you know about fetal movements as the pregnancy progresses towards childbirth?***	The movements increase	16 (57.1)
	They remain the same	8 (28.6)
	The movements decrease	8 (28.6)
	I don’t know	0
	Other	0
***How do you advise women to monitor the way their baby moves?***	Tell them to be aware of the movements of their baby	21 (75.0)
	Tell them to count the movements	10 (35.7)
	Other: ‘Monitor the timing when fetal movement increases’	2 (7.1)
	I don’t tell them about monitoring fetal movements	0
***Do you have any advice for women concerning actions they can undertake when they don’t feel their baby move as usual and are worried?***	To come to the hospital	26 (92.9)
	Lie down, rest, and focus on the movements	7 (25.0)
	To ask for advice from their husbands and relatives	1 (3.6)
	Other: ‘To do ultrasound scan’	1 (3.6)
	No, I don’t	0
	Act as usual, don’t undertake any action	0

#### General awareness of FMs

*Pregnant and postpartum women*. All ANC women remembered the first time they felt their baby move and the pattern of their baby’s movements in the recent days. Participant descriptions of FM patterns were variably associated with time-of-day, maternal activity and in many cases environmental stimuli such as maternal hunger, eating, maternal position or touching the abdomen.

*‘Normally*, *I feel it kicking mostly after eating*, *in the morning and in the afternoon*. *When I’m resting the baby starts kicking*…*I know what’s normal for my baby.’*

*(Antepartum)*

*‘With the stillbirth I had before*, *it happened that I was worried then*. *Now I’m even more alert.’*

*(Antepartum)*

*Healthcare providers*. All health providers understood FMs that was line with the following:

*‘Fetal movement (‘uchezaji wamtoto’) is the movement felt by the mother during pregnancy*, *which normally starts to be felt from the 16*^*th*^ *week of gestation age until the time of delivery.’*

*(Healthcare provider)*

#### Interpretation of fetal movements and their abnormal patterns

*Pregnant and postpartum women*. All women interviewed in the ANC considered FMs as a sign of life of the fetus and a form of communication between fetus and mother.

‘*When the baby is kicking*, *I know it’s continuing in good health*. *It’s doing well’*

*(Antepartum)*

*‘When I’m too busy the baby will let me know by kicking me once very strongly*. *That’s how it tells me to slow down*. *Normally the baby is quiet at night*, *but when I’m lying on one side for too long the baby will kick strongly*, *as if to tell me*, *or reminds me ‘Hey*! *Turn the other side!”*

*(Antepartum)*

Half the ANC women perceived FMs as abnormal, or worrisome, if they become absent. Other women found FMs abnormal if they become weaker or less frequent than usually.

*‘If the baby is not kicking*, *I will be worried (…)*. *Because maybe the baby already started to die’*

(Antepartum)

Furthermore, women also perceived FMs and their changes during labour:

*‘When my blood pressure was high [250/120 mmHg]*, *I felt the baby was moving differently*. *It moved slower than before that*. *During labour also it sometimes stopped moving which worried me.’*

*(Postpartum)*

*‘At home it [FMs] was just fine*, *but since I arrived*, *I felt it became less and less*. *I already started to drain [losing amniotic fluid] at home but once I arrived here*, *I drained more and after that the movements became even weaker’*

*(Postpartum*, *baby died after delivery)*

*Healthcare providers*. All health care providers also considered the presence of FMs as a sign of health of the fetus and mother. Reported worrying changes included reduced, absent and increased FMs. (Table 2) Various causes of abnormal FMs were given such as maternal as well as fetal conditions such as fetal maternal emotions, activity, hunger, intake of herbal medication, anemia, infection, hypertension and bleeding, position and sleep state of the baby, polyhydramnios and oligohydramnios. Pointing to a poster on the wall, an ANC nurse also indicated that abnormal FMs is one of the danger signs they look out for.

*‘To ask a mother whether her baby moves or not helps us to decide whether it’s alive or not*. *They [FMs] may be increased or decreased which will help us to know whether the baby is healthy or not*.

*(Healthcare provider)*

### Behavior and practice

#### Monitoring and assessment of FMs

*Pregnant and postpartum women*. All ANC women stated they instinctively knew when something was wrong with their baby through the way the fetus moves. None of the women in the ANC said they monitored FMs by means of writing down or counting.

*‘I don’t monitor; I just know when something is wrong*. *I recognise every change.’*

*(Antepartum)*

Postnatal women remembered being asked about FMs on admission to the labour ward either by a research assistant as part of this study or by routine nurse-midwives. However, the minority of women had FMs findings documented in their hospital files.

*Healthcare providers*. Staff assessed FMs by asking women whether the baby is playing (‘*anacheza’*) or kicking (‘*anapiga*’), by palpation of the abdomen and/or by ultrasound. Nearly all staff responded that they ‘*almost always*’ or ‘*often’* assess FMs when assessing women in the clinic, on admission and during labour (Table 2). However, answers were variable during interviews and FGDs: the two nurses in the regular ANC said they always assessed FMs, and staff in the obstetric clinic stated they assessed FMs only if there was a complaint from the mother. Yet, the majority of women told they were never asked about FMs at the ANC clinics of MMH. Nurse-midwives were almost unanimous in reporting that they always ask mothers about FMs on admission to the labour ward. Although many claimed they assessed timing and perceived changes in FMs, only information about the presence or absence of FMs was documented in patient files.

*‘You ask the mother ‘How are your baby’s movements today*?*’ and will say ‘It’s normal or today the playing increased it decreased or I didn’t feel the baby move today*.”

*(Healthcare provider)*

*‘*…*when we assess the mother and find no fetal heart rate we always ask about fetal movement to confirm fetal condition of health and viability’*

*(Healthcare provider)*

Opinions were divided regarding assessment of FMs during labour. Amongst nurse-midwives it appeared not to be routine practice.

*‘In the ward we don’t ask because even if you can ask*, *the mother cannot give the true answer while in labour*. *She may say that she doesn’t know if there are movements because of the labour pain…*. *we just monitor fetal heart rate but movements we almost never assess during labour.’*

*(Healthcare provider)*

Half of the registrars said they assessed FMs during labour, however the rest did not agree and elaborated:

*‘We normally don’t ask in the labour ward*, *honestly*. *When we check the admission form and we find a [positive] FHR then we just monitor these women.’*

*(Healthcare provider)*

*‘Or when there is bleeding*, *you ask them’*

*(Healthcare provider)*

#### Management of abnormal maternal perception of fetal movements

*Pregnant and postpartum women*. During pregnancy, women said they would eat, rest, massage the abdomen and wait if they get worried about the way their baby was moving. For how long they would wait before seeking health care differed from a few hours, to more than 24 hours or up to two weeks. All women who said they would present within a few hours had received health education in peripheral ANC clinics. A minority of the ANC women mentioned they had a history of seeking medical help because of abnormal FMs and wanted a fetal ultrasound. One woman in the ANC said she went to a midwife in her community for an abdominal massage.

‘*When the baby will stop moving or I’m worried about it*, *I will not directly come to the hospital*. *First*, *I will wait and see what the situation is*. *If it’s still not moving after 2 days*, *I will come to the hospital*. *I’m afraid to directly come to the hospital because maybe I will get shocking information or they will send me home because it was nothing*. *I prefer to wait and see’*

*(Antepartum)*

Asking for advice from relatives, their husband, friends or neighbors in case of worrisome FMs was mentioned by women in the ANC.

‘*I was concerned on admission*, *but actually 3 days before already*, *my baby had stopped moving*. *I asked my neighbor about this*, *she told me it was normal for the baby to stop moving towards term*. *So*, *I didn’t worry that much*. *I also asked my mom whether it was normal for the baby to stop*, *but she didn’t remember it anymore because it was too long ago she was pregnant herself*. *She could not advise me on this’*

*(Postpartum*, *delivered stillborn baby)*

*Healthcare providers*. Midwives stated that they will first assess a mother’s mental, physical and social health and lifestyle because these all can contribute to abnormal FMs. All health providers stated they advise women to stimulate their baby with various means in case of absence of FMs: to exercise, drink cold water, eat something (sweet), change position or rest. Moreover, the staff unanimously responded that women should go to nearby clinic or hospital ‘*immediately’* or ‘*as soon as possible*’, ‘*even during the night*’ in case of worrisome changes in FMs.

‘*You can save a baby or lose a baby in 1 hour*, *so why waste any time*? *Why wait*?’

*(Healthcare provider)*

Management of decreased or absent FMs at the clinic or hospital consisted of auscultation of fetal heart rate (FHR) and performing obstetric ultrasound. Nurse-midwives commonly evaluated the mother and fetus to rule out problems and reassure mothers while registrars doctors usually suspected intrauterine fetal death. Women were advised, reassured and sent home if fetal viability was confirmed or they proceeded to further management in case of an intrauterine death or other complications.

‘*First*, *I will ask the mother what happened before I can think of what caused the changes in FMs*. *Then we do an ultrasound and advise the mother to change position*, *especially if the ultrasound is normal*. *If it is abnormal*, *we have to take action accordingly’*

*(Healthcare provider)*

*‘When a woman for example comes to the hospital in the evening and complains about reduced fetal movements or that the movements have been absent for a couple of hours*: *you check FHR and you confirm [fetal] death’*

*(Healthcare provider)*

*‘*…*we check FHR and if it’s there and there are no other danger signs*, *we tell her to come back in 2 weeks…*. *Why not observe her here if there’s any change in fetal movements*?’

*(Healthcare provider)*

### Barriers to practice

#### Lack of knowledge and education

*Pregnant and postpartum women*. Some women, especially those who attended antenatal classes in peripheral ANC clinics, were told to monitor FMs. However, they were never told or taught *how* to monitor FMs by health providers. Women reported being asked about FMs during ANC and labour particularly when there was a medical problem (e.g. hypertension) or absence fetal heart rate. Some postnatal women said they did not know anything about the importance of FMs or what to do when changes were perceived.

*‘Before and on admission my baby was moving less and less*. *During labour the baby stopped moving*, *I did not feel it anymore*. *What should I do next time when it stops moving*?’

*(Postpartum*, *delivered a stillborn baby)*

#### Inability to always express concerns about perceived abnormal fetal movements

*Pregnant and postpartum women*. Many postpartum women said that they experienced worrying reduced or absent FM by the time of admission and/or during labour care. We confirmed through our main study [16] that many of these women reported abnormal FMs on admission but, according to the documentation, some women did not report their concerns. Fewer women said that they expressed their concerns about FMs during labour. Reasons for not reporting concerns of FMs include: women thought it was not important, they did not feel free, they were not asked, or the doctor was too busy. The majority of women who said they had perceived abnormal FMs on admission and during labour care had adverse perinatal outcomes. Other women had pre-eclampsia and delivered vaginally with healthy babies.

*‘During labour I was very worried because I didn’t feel the baby moving the way I was used to*. *In the evening the baby totally stopped moving and then I got an ultrasound scan*. *The baby was already dead when they did the ultrasound scan*. *Before the operation I heard it wasn’t alive anymore’*

*(Postpartum*, *delivered a stillborn baby)*

‘*This is my first child and I didn’t know anything about pregnancy or how my baby should be moving*. *Before I was in labour my baby was moving just fine*. *When I got into labour*, *it was moving very slowly*. *I didn’t tell the doctor because I didn’t know anything about it*. *I was worried because I wasn’t experienced*: *I never delivered before’*

*(Postpartum*, *baby died after birth)*

Additionally, both nurse-midwives and registrar doctors stated that women rarely present at the hospital because of decreased FMs or present if it is too late (e.g. the fetus has died or there are serious obstetric complications). If women are not asked, they will not express their concerns.

*‘Mothers who come here don’t tell you ‘I haven’t felt my baby kicking for 4 hours’*. *They tell you*: *‘I haven’t felt my baby move since yesterday*, *or longer*”

*(Healthcare provider)*

‘*Yes*, *it does happen that women express concerns about the way their baby is moving*. *There are some mothers that are really concerned about their baby*. *They come to you and tell you that their baby is moving differently*. *Not all mothers*, *just a few*. *Very few’*

*(Healthcare provider)*

#### Staff perception of women’s awareness of FMs

*Healthcare providers*. In all FDGs, health providers agree that the best judge about FMs patterns is the mother herself. However, they were convinced that women did not have enough awareness and knowledge about FMs, do not understand questions about the way their baby is moving, and therefore asking them about it does not contribute to their care.

‘*Only if the mother does routinely check the FMs*, *it’s possible to know if the baby is asleep or not*. *She has to know the rhythm of the baby*. *Most mothers don’t know the pattern of their baby’s movement and they don’t really know whether the baby is kicking or asleep’*

*(Healthcare provider)*

‘*Some mothers are asked and know about changes but most do not understand’*

*(Healthcare provider)*

‘Primes don’t really know what a kick or a push is, so you have to explain to them how it feels’

*(Healthcare provider)*

ANC nurses said to trust women’s perception of FMs.

*‘I think it is worrisome when it’s [FMs] abnormal for the mother*. *A mother knows when something is wrong with her child*. *So*, *if she says it [FMs] is different and she’s worried*: *it is*’.

*(Healthcare provider)*

#### Limited resources for teaching and assessment: Space and staff

*Pregnant and postpartum women*. The timing of antenatal classes was a problem for four women who attended ANC clinics elsewhere:

*‘I wasn’t taught about it*, *but I know that in the nearby clinic they teach women about it [FMs]*. *But I always come late for this*, *since they start around 7am with the class’*

*(Antepartum)*

*Healthcare providers*. The main problem for the ANC nurses when it comes to educating women, was lack of space to hold antenatal classes. All registrars and nurse-midwives stated that the high volume of women and too few staff were problems for assessing FMs during admission and labour care. They suggested an extra nurse and more room for admission.

‘*As my colleagues said*, *sometimes due to overcrowding of women on arrival the nurse forgets to ask some details to the mother.’*

*(Healthcare provider)*

‘*Also we need time*, *adequate number of staff*, *especially in the admission room*.*’*

*(Healthcare provider)*

*‘Most of the time*, *we don’t pay much attention to low-risk women unless there is a problem*. *We prioritise women*. *For high-risk women we ask everything*. *But low risk*: *no’*

*(Healthcare provider)*

#### Attitude and habit

*Healthcare providers*. The most common reason for staff not to ask about FMs was that it was just not routine practice, although they said it may not take much time and could be done while performing physical examination.

*‘It’s a habit*, *it’s not our routine to ask women about it*. *We are used not to ask’*

*(Healthcare provider)*

‘*I actually think it’s just a habit*. *…We never do it*, *although it only costs one minute to go into the low-risk labour ward and ask ‘Which women did not feel their baby move today*?*’*. *But we don’t do that*, *because that’s the habit’*

*(Healthcare provider)*

### Opportunity for improvement

#### Education and guidance

All the interviewed participants, women, and staff responded positively towards more education and training about maternal perception of FMs. ANC nurses mentioned they want to have antenatal classes to educate women about pregnancy, if they get another room for this.

*‘Yes*, *I would like to have a course about it so I learn more about what it is and about what to do*. *I wish the public to be taught because otherwise we just sit at home and wait’*

*(Antepartum)*

A simple, short standard questionnaire was also suggested to improve assessment in both the ANC, on admission and intrapartum.

*“If we change the partogram and add a question about FMs*, *maybe things will change because it can remind them about asking the patient*.”

*(Healthcare provider)*

Several nurse-midwives suggested to have guidelines for assessment and management:

*‘I think we should have a chart*, *a short questionnaire posted on the wall of the admission room to remind the midwife the importance of FMs*, *how to assess and what actions to take with the findings.’*

*(Healthcare provider)*

## Discussion

### Main findings

This mixed-method study showed that women and their healthcare providers in a low-resource setting have high awareness of maternal perception of fetal movements. Pregnant women instinctively knew the unique normal and abnormal patterns of their baby’s movements, both in the antepartum and intrapartum period more than assumed by their health providers. However, there was a lack of acquired knowledge and guidance of maternal perception of FMs and how to use this information by both women and staff alike for routine fetal surveillance and management of reduced FM. This led to unexpressed concerns, delayed presentation with reduced FMs and missed opportunities to save lives. The high volume of women and the lack of (human) resources at the maternity unit was an important finding that prevents healthcare providers from educating women and asking them about FM. These results provide an opportunity to increase awareness and knowledge through more education of pregnant women and health providers and to jointly develop a locally applicable protocol for reduced FMs management.

### Strengths and limitations

This is the first study that assessed women and their healthcare providers’ awareness, knowledge, and practices on maternal perception of fetal movement in low- and middle-income countries (LMICs). It benefited from triangulation of quantitative and qualitative methods, however, to improve understanding, the scope could have been widened to include the views of FMs from specialised/senior healthcare providers and the wider community. As the study is limited to one hospital and the results based on the researchers’ subjective interpretation, the findings are not generalizable and cannot be assumed to represent the general awareness, knowledge and practices regarding FMs in other LMICs settings. Also, the questionnaires used may have been biased towards agreeable responses and might not reflect true opinions and practices, hence the usefulness of FGDs.

### Interpretations

Of the 2.6 million pregnancies that end in stillbirth, the overwhelming majority occur in LMICs. However, the evidence to guide optimal fetal monitoring is limited. Although strong evidence suggest that reduced fetal movement is associated with intrauterine fetal death, it remains unclear whether it is a symptom of inevitable fetal death or whether it can be used as an alert to prompt action and improve outcome [21, 22]. This study showed that FMs assessment during labour, including on admission to the labour ward may be important as many women who prospectively and retrospectively reported abnormal FMs during labour had subsequently developed perinatal adverse outcomes.

The little evidence available in LMIC indicates that women’s awareness of the importance of FMs and reduced FMs as a danger sign of adverse pregnancy outcomes was shown to be low and varied from 3.1% to 62.3% across LMICs; and no studies were found to assess healthcare providers’ knowledge of FMs [23]. In this study setting, it was found that all women were aware of the unique patterns of FMs—supporting an individualised definition of abnormal/reduced FMs in accordance with the perception of the mother. Also, most women and all their healthcare providers knew the importance and interpretations of FMs as a sign of health of the child and mother. However, there were no assessment of FMs or management guidelines for women presenting with reduced fetal movement which also reflects the lack of evidence and international consensus [22].

Although there has been recent interest in maternal perception of FMs and evidence is being sought in large trials in high-income countries, there is no single RCT in settings with the highest burden [15]. Recent trials in HICs showed educational and management packages or FMs awareness intervention did not improve perinatal outcomes [21, 24]. However, awareness of fetal movement is already incorporated in stillbirth reduction strategies in HIC and stillbirth are much less common. We can assume, therefore, that the high baseline knowledge of FMs of both women and health provider and the already-existing protocols on reduced FMs minimises the effect of new or specific package of intervention being tested. Thus, the current evidence might not be readily applicable to settings with lower awareness and knowledge and no established guidelines for assessment and management of abnormal FMs. The role of maternal perception of fetal movement as part of a stillbirth prevention strategy needs to be explored in these settings where the stillbirth rates are much higher. It is especially relevant in low-resource settings like MMH, where human resource is scarce and the workload is considerably high, with little time for adequate fetal heart monitoring, that an educated, alert and involved woman might help improve her own care. Whether formally or informally monitored, FMs can be the *only* signal of fetal compromise in absence of regular antenatal and intrapartum checks especially in high-risk pregnancies.

Whether or not fetal movement monitoring is advised or encouraged, some women did have concerns about fetal movement. They should be able to express their concerns and healthcare providers should be equipped with evidence-based knowledge and practice to address them. However, in this study, women lacked the agency to express concerns and staff did not always assess FMs due reasons related to attitude/unfamiliarity with assessing them, workload, lack of (human) resources and staff misconception and lack of trust of women’s perceptions and knowledge of FMs. Trust in women’s perception of FMs was even less during labour where the general view is that labour pain obscures maternal perception of FMs. The potential benefits and harms of FMs monitoring and interventions, including unnecessary maternal anxiety and obstetric interventions, prematurity, hospital admissions, and increased work overload for staff in an already overwhelmed system, makes it a priority area for research [16].

## Conclusion

This study shows that women and their healthcare providers in a low-resource setting have high awareness of maternal perception of fetal movements, but little use of FMs assessment in routine clinical practice to improve care. Thus evidence-based monitoring and management guidance for reduced FMs is essential and an opportunity to prevent adverse outcomes in low-resource settings.

## Supporting information

S1 Text Consolidated criteria for reporting qualitative studies (COREQ): 32-item checklist. (DOCX)

S2 Text Questionnaires interviews women + guideline FGDs health providers. (DOCX)

S3 Text Questionnaire health providers. (DOCX)
