# Supplementary figures and images for: Maternal perception of fetal movements: Views, knowledge and practices of women and health providers in a low-resource setting
Source: PLOS Glob Public Health. 2023 Mar 29;3(3):e0000887. doi: 10.1371/journal.pgph.0000887 (PMC10058116; doi:10.1371/journal.pgph.0000887)

**S1 Text: Consolidated criteria for reporting qualitative studies (COREQ): 32-item checklist.(11)**


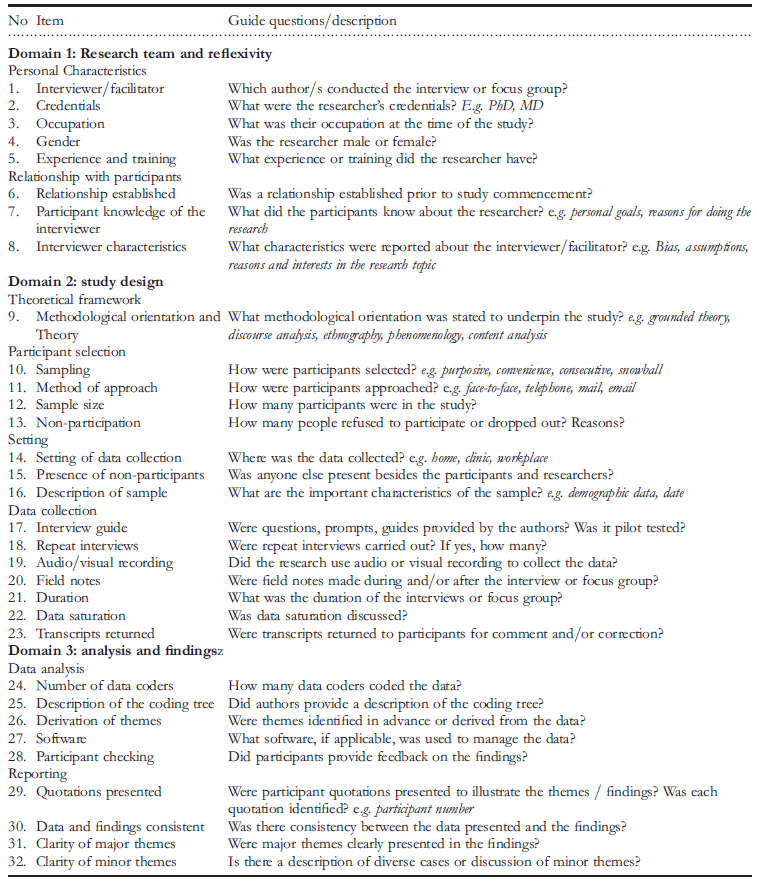

Supplement: S1 Text — (DOCX) [file pgph.0000887.s001.docx]
