# Supplementary material for: Maternal perception of fetal movements: Views, knowledge and practices of women and health providers in a low-resource setting
Source: PLOS Glob Public Health. 2023 Mar 29;3(3):e0000887. doi: 10.1371/journal.pgph.0000887 (PMC10058116; doi:10.1371/journal.pgph.0000887)
Supplement: S2 Text — (DOCX) [file pgph.0000887.s002.docx]

**S2 Text: Questionnaires interviews women + guideline FGDs health providers**

**Interviews women: directive ANC**

Definition of foetal movements:

1. In this pregnancy, have you felt your baby kick? If yes, when did it start?
2. Could you describe the way your baby usually moves? (If no clear answer, see A-E)
3. How do the movements change throughout the day?
4. How many times per day does your baby move?
5. Are the movements strong or weak?
6. When does the baby move the least?
7. When does the baby move the most?
8. If multiparity: do the movements of this baby differ from previous pregnancies? If yes, how?
9. What do the movements of your baby tell you?
10. When would you be worried about your baby’s movements? Did you ever experience this?

Education on foetal movements:

1. Who taught you about foetal movement? What did they say?
2. How do you monitor your baby’s movement? Why do you do it the way you do it?
3. Are you being told about how to monitor your baby’s movements in ANC?
4. Do you get asked about your baby’s movements when you visit ANC?
5. Do you get any information about what do when you are worried about the way your baby moves? If so what advice do you get?
6. Do you want to receive information about foetal movements in the ANC? And why?
7. Do you talk about foetal movements with other people? (mother, other relatives, friends) e.g. do they advise you on this?

Management of changes in foetal movements:

1. What would you do if there are changes in foetal movement? (e.g. change position, rest, lie down, eat something, wait, seek for traditional help)
2. What would you do if these changes worry you? (e.g. change position, rest, lie down, eat something, wait, seek for traditional help, go to the hospital)
3. Have you ever sought health care because of reduced or other changes in movements? (also can be in previous pregnancies)

**Interviews women: directive postpartum**

Foetal movements on admission and during labour:

1. When you came to the hospital for admission, did you get asked about the way your baby was moving?
2. If yes, were you able to answer all the questions?
3. Did you understand the questions being about your baby’s movement?
4. If not, what didn’t you understand?
5. Did you have any concerns about your baby based on the way it was moving, before or during admission?
6. If you expressed any concerns, what happened?
7. Did you feel free to express these concerns during admission, about the way your baby was moving? Is there anything that worried you that you were not asked or you could not express?
8. During labour in the hospital, were there any other changes in the way your baby was moving that worried you? If yes, what changed?
9. If yes, were you able to express them to the staff in the wards? What happened when you expressed them?
10. Is there anything else we could have asked you about the way your baby moved?

**Directive FGDs + semi-structured interviews health providers**

1. What questions do you ask women when you assess their perception of foetal movements? Please also tell what Kiswahili words you use.
2. What, in your opinion, do foetal movements tell us about the baby? (Please try to explain in detail)
3. What do you know about foetal movements as pregnancy progresses towards childbirth? Do they remain the same as before or change?
4. What do you think are the most worrying changes in foetal movement? And why?
5. What things do you think can cause changes in foetal movements?
6. Do you advise women to monitor the way their baby moves? And if yes, **how** do you advise women to monitor the way their baby move?
7. Do you have any advice for women concerning actions they can undertake when they don’t feel their baby move as usual? (Please also ask for traditional ways to remedy/arouse foetal movements)
8. When do you advise women to come to the hospital in case the movements of their baby changed? (Hours? Days?)
9. Do you ask women about foetal movements in the ANC, admission room and labour room?
10. If yes, does it happen that women express concerns about the way their baby is moving?
11. How do you treat/manage a woman who presents with decreased or absent foetal movements or express their concerns because their baby moves differently?
12. What could be a barrier for not asking women about foetal movements?
13. If you believe that asking women about foetal movements is not done adequately at MMH, what could be the reasons for this?
14. Could you suggest possible ways to improve foetal movement assessment in the ANC, on admission and intrapartum at MMH?
15. Is there anything else you want the researcher to know or if you have comments and/ or suggestions ?
