# Supplementary material for: Maternal perception of fetal movements: Views, knowledge and practices of women and health providers in a low-resource setting
Source: PLOS Glob Public Health. 2023 Mar 29;3(3):e0000887. doi: 10.1371/journal.pgph.0000887 (PMC10058116; doi:10.1371/journal.pgph.0000887)
Supplement: S3 Text — (DOCX) [file pgph.0000887.s003.docx]

**S3 Text: Questionnaire health providers**
